# Expression pattern of *Stlhcb* gene family in potato and effects of overexpression of *Stcp24* gene on potato photosynthesis

**DOI:** 10.1371/journal.pone.0305781

**Published:** 2024-08-23

**Authors:** Xinhua Tang, Yulin Liu, Shiwei Li, Yating Pei, Qiaorong Wei, Lili Zhang, Ying Shi

**Affiliations:** 1 College of Agriculture, Northeast Agricultural University, Harbin, China; 2 National Research Center of Soybean Engineering and Technology, Harbin, China; 3 Key Laboratory of Germplasm Enhancement and Physiology & Ecology of Food Crop in Cold Region, Ministry of Education, Harbin, China; Hainan University, CHINA

## Abstract

Potato is one of the four staple food crops in the world. It has a wide range of cultivation, high yield, and high nutritional value. Enhancing the photosynthesis of potato is particularly important as it leads to an increase in the potato yield. The light-harvesting pigment-binding protein complex is very important for plant photosynthesis. We identified 12 *Stlhcb* gene family members from the potato variety "Atlantic" using transcriptome sequencing and bioinformatics. The proteins encoded by the *Stlhcb* gene family have between 3358 and 4852 atomic number, a relative molecular weight between 24060.16 and 34624.54 Da, and an isoelectric point between 4.99 and 8.65. The RT-qPCR results showed that the 12 *Stlhcb* genes were expressed in a tissue-specific and time-dependent fashion under low light. The relative expression of the *Stlhcb* genes in the leaves was significantly higher than that in the stems and roots, and the relative expression of these genes first increased and then decreased with the prolongation of light exposure time. The *Stcp24* gene with the highest expression was cloned, and an expression vector was constructed. A subcellular localization analysis was performed in tobacco and an overexpression experiment was performed in potato using an Agrobacterium-mediated method. The subcellular localization analysis showed that the protein encoded by *Stcp24* was located in chloroplasts as expected. Overexpression of *Stcp24* in transgenic potato increased the yield of potatoes and the content of chlorophyll a and b; increased the net photosynthetic rate, transpiration rate, stomatal conductance, electron transport efficiency, and semi-saturated light intensity; and promoted photosynthesis and plant growth. This study provides a reference for the study of the function of the potato light-harvesting pigment-binding protein gene family. It lays a foundation for further study of the mechanism of the photosynthesis of potato, improvement of the light energy utilization of potato, and molecular breeding of potato.

## 1 Introduction

Potato (*Solanum tuberosum* L.) is one of the four staple food crops in the world. It has high nutritional value and high yield and is the third most important food crop in the world [1]. Potato is a staple food for approximately 30% of the world’s population [2]; thus, a high and stable yield of potato plays an important role in ensuring world food security [3]. The organic compounds synthesized by photosynthesis in potato leaves are transported to underground tubers in the form of sucrose for decomposition, energy supply, or storage. Enhancing the photosynthesis of potato is particularly important as it leads to an increase in the potato yield. Photosynthesis consists of light reactions and dark reactions. Among them, the light reaction must be accomplished through a series of pigment–protein complexes on the photosynthetic membrane, in which the key step is the energy conversion caused by the light-induced charge separation that takes place in the reaction center.

At present, there are few studies on the function and mechanism of the *Lhcb* gene in potato; thus, further study of the *Lhcb* gene in potato is of great significance in the improvement of the photosynthetic efficiency and energy acquisition of potato. In this study, transcriptome sequencing combined with bioinformatics analysis and RT-qPCR technology was used to analyze the transcriptional level and spatio-temporal expression pattern of the potato light-harvesting pigment-binding protein gene under low light stress.

The *Stcp24* gene of the potato *Stlhcb* gene family was cloned, and the stable genetic transformation of potato and subcellular localization studies in tobacco were carried out. The growth index, photosynthetic index, and fluorescence kinetic parameters were determined.

This study elucidates the function of the potato *Stcp24* gene. Saving light energy in the process of the detoxification of potato and improving the utilization rate of light energy by intercropping with high-stem crops are of great significance. These processes are expected to expand the potato planting area and allow the potato industry to improve its quality and efficiency; they are also expected to lay a foundation for an in-depth study of potato photosynthetic function and molecular breeding to improve potato photosynthetic efficiency.

## 2 Materials and methods

### 2.1 Test materials and treatment

Our previous study found that the potato variety "Atlantic" was more tolerant of low light [4]. Therefore, it was selected for this study. Tissue culture seedlings were propagated and cultured in a light incubator for 30 d (16 h/8 h light/dark, light intensity 56 μmol·m^-2^·s^-1^, 24°C). Then, several seedlings with uniform growth were selected and treated under low light intensity (16 h/8 h light/dark, light intensity 11 μmol·m^-2^·s^-1^, 24°C). The third and fourth leaves of the upper part of the plant were taken at 0 h, 24 h, and 48 h of treatment and frozen in liquid nitrogen. The leaf samples were collected and stored at -80°C for transcriptome sequencing. All of the biological replications were carried out three times.

### 2.2 Transcriptome sequencing and data processing

The transcriptome was sequenced on the high-throughput sequencing platform (Illumina HiSeq 2000, USA) of Beijing Baimaike Biology Co., Ltd. Transcriptome data uploaded to the Integrated Gene Expression Database (GEO).The differentially expressed genes were analyzed using the Bemaker online data analysis platform (www.biocloud.net), and the significant P value obtained from the original hypothesis test was corrected using DESeq software and the Benjamini–Hochberg method. The corrected P value, that is, the error detection rate (false discovery rate, FDR) < 0.05, was used as the key index for the screening of the differentially expressed genes in order to reduce the false positives caused by the independent statistical hypothesis test for the expression value of a large number of genes. FDR < 0.05 and |log2FC| > 1 (fold change, FC) were used as the filtering criteria. The differentially expressed genes were analyzed using GO enrichment analysis and KEGG pathway enrichment analysis.

### 2.3 Bioinformatics analysis

The physical and chemical properties of the light-harvesting pigment-binding proteins were analyzed using the ExPASy online website (https://web.expasy.org/protparam/) [5]. The hydrophobicity of the light-harvesting pigment-binding proteins were analyzed using an online website (https://web.expasy.org/cgi-bin/protscale/) [6]. The secondary structure of the light-harvesting pigment-binding proteins were predicted using an online website (https://npsa-prabi.ibcp.fr/cgi-bin/) [7]. The tertiary structures of the light-harvesting pigment-binding proteins were predicted using an online website (https://www.swissmodel.expasy.org/) [8]. The subcellular localizations of the light-harvesting pigment-binding proteins were predicted using an online website (http://linux1.softberry.com/berry) [9]. The multi-sequence alignment of the *Lhcb* gene in potato, tomato, tobacco, and pepper was carried out using MEGA11.0 software; then, the common phylogenetic tree of the *Lhcb* gene in potato, tomato, tobacco, and pepper was constructed using the maximum likelihood method. The bootstrap method was used and was set to repeat 1000 times.

### 2.4 RT-qPCR for gene expression analysis

The tissue culture plantlets of the potato variety "Atlantic" were cultured in a light incubator for 30 d (16 h/8 h light/dark, 56 μmol·m^-2^·s^-1^, 24°C) and placed in 100% (56 μmol·m^-2^·s^-1^ control)), 60% (34 μmol·m^-2^·s^-1^), and 20% (11 μmol·m^-2^·s^-1^) incubators, respectively. The leaves, stems, and roots were taken at 0 h, 4 h, 8 h, 12 h, 16 h, and 20 h, and the leaves, stems, and roots were frozen in liquid nitrogen and stored at -80°C. All of the biological replications were carried out three times.

Based on the results of the transcriptome sequencing of the potato variety "Atlantic", 12 differentially expressed light-harvesting pigment-binding protein genes were obtained, and real-time quantitative PCR primers were designed using Primer5.0 software (S1 Table in S1 Data). The expression of the internal reference gene (ef1α) was used as a control in the real-time quantitative PCR tests. The RNA extraction method was that used in the study by Tang et al. [10]; this method uses the HiFiScript gDNA Removal cDNA synthesis kit and a reverse transcription kit (Kangwei Company, Beijing) to synthesize single-stranded cDNA. In accordance with the instructions of the ULtraSYBR mixture kit (Kangwei Company, Beijing), the reaction system was configured as follows: cDNA 2 μL; positive and negative primers (10 μmol/L) 0.5 μL; 2 × UltraSYBR Mixture 10 μL; ddH_2_O 7 μL; and total volume 20 μL. The reaction was pre-denatured at 95°C for 10 min, denatured at 95°C for 10 s, annealed at 52°C, and extended for 32 s at 72°C for 35 cycles. The melting section was 95°C for 30 s, 52°C for 1 min, and 95°C for 30 s. The expression of the internal reference gene and target gene was detected using a real-time quantitative PCR instrument (Line Gene9620, China). All of the biological replications were carried out three times. The relative gene expression was calculated using the 2^-ΔΔCt^ method.

### 2.5 Subcellular localization

*Agrobacterium tumefaciens* containing the pCAMBIA1300 vector and *A*. *tumefaciens* containing the pCAMBIA1300-CP24 vector were injected into Ben’s tobacco leaves with a 1 ml aseptic syringe. The bacterial solution was fully permeated, and the tobacco was subjected to dark treatment in a 24°C incubator for 48 h. The fluorescence signal of GFP was detected using a confocal laser scanning microscope (TCS SP8 Leica, Germany) at 488 nm wavelength.

### 2.6 Cloning and vector construction of *Stcp24* gene of light-harvesting pigment-binding protein in potato

The RNA from the potato variety "Atlantic" was extracted using the TRIzol method and then reverse-transcribed into cDNA using a kit. Using the cDNA as a template, the CDS sequence of *Stcp24* was amplified with CP24-F/R primers (excluding the stop codon) (S1 Table in S1 Data). The *Stcp24* gene was ligated into the pCAMBIA1300 plant overexpression vector using double-enzyme digestion. The plasmid was amplified in *Escherichia coli*, extracted and sequenced, and then transformed into GV3101 Agrobacterium competent cells.

### 2.7 Acquisition and identification of transgenic potato

The *Stcp24* gene was transformed into the potato variety "Dongnong 310" by *A*. *tumefaciens*, and transgenic plants were obtained. A plant genomic DNA extraction kit (Kangwei Co., China) was used to extract genomic DNA from the leaves of the regenerated potato seedlings. The primer 35S+CP24F/R (S2 Table in S1 Data) was designed based on the *Stcp24* gene sequence and its front-end vector sequence. The extracted DNA was used as a template, and 35S+CP24F/R was used as a primer for PCR identification. RNA was extracted from the leaves of the transgenic plants and reverse transcribed into cDNA. Real-time fluorescence quantitative PCR was performed using CP24F/R (S2 Table in S1 Data) as a primer to verify the transgenic plants.

### 2.8 Determination of the physiological indexes of transgenic potato

The transgenic seedlings with uniform growth and the control were propagated in an MS solid medium using stem cutting and cultured in a light incubator for 25 d. The culture temperature was 24°C, and the light intensity was 56 μmol·m^-2^·s^-1^ (16 h/8 h, light/dark). Then, they were transplanted into pots with coconut shell and peat (coconut shell:peat/2:1) as substrates. The data were measured in a greenhouse with a culture temperature of 24°C and a light intensity of 131 μmol·m^-2^·s^-1^ (16 h/8 h, light/dark) for 30 d. The relative chlorophyll content of the third and fourth leaves of the upper part of each test plant was measured using a chlorophyll meter (SPAD-502, Japan) [11]. The fluorescence parameters were measured using a chlorophyll fluorescence spectrometer (PAM-2500, Germany), and the leaves were acclimated in the dark for 20 min before the measurements [12]. The photosynthetic indexes, including net photosynthetic rate, transpiration rate, stomatal conductance, and intercellular CO_2_ concentration, were measured using a portable photosynthesis system (CI-340, USA) [13]. The contents of chlorophyll a and b were determined using spectrophotometry (P-Class330, Germany) [14]. After the leaves were completely brown, the potatoes were harvested and the yield was measured. All of the biological replications were carried out three times.

### 2.9 Statistical analysis of data

Student’s t-test was performed using SPSS software (v.23.0) to determine significance, which was defined as P < 0.05 (*), P < 0.01(**). Graphpad 7 and TBtools software were used for plotting [15].

## 3 Results

### 3.1 Transcriptome sequencing analysis

#### 3.1.1 Sequencing quality and results

**Fig 1:**
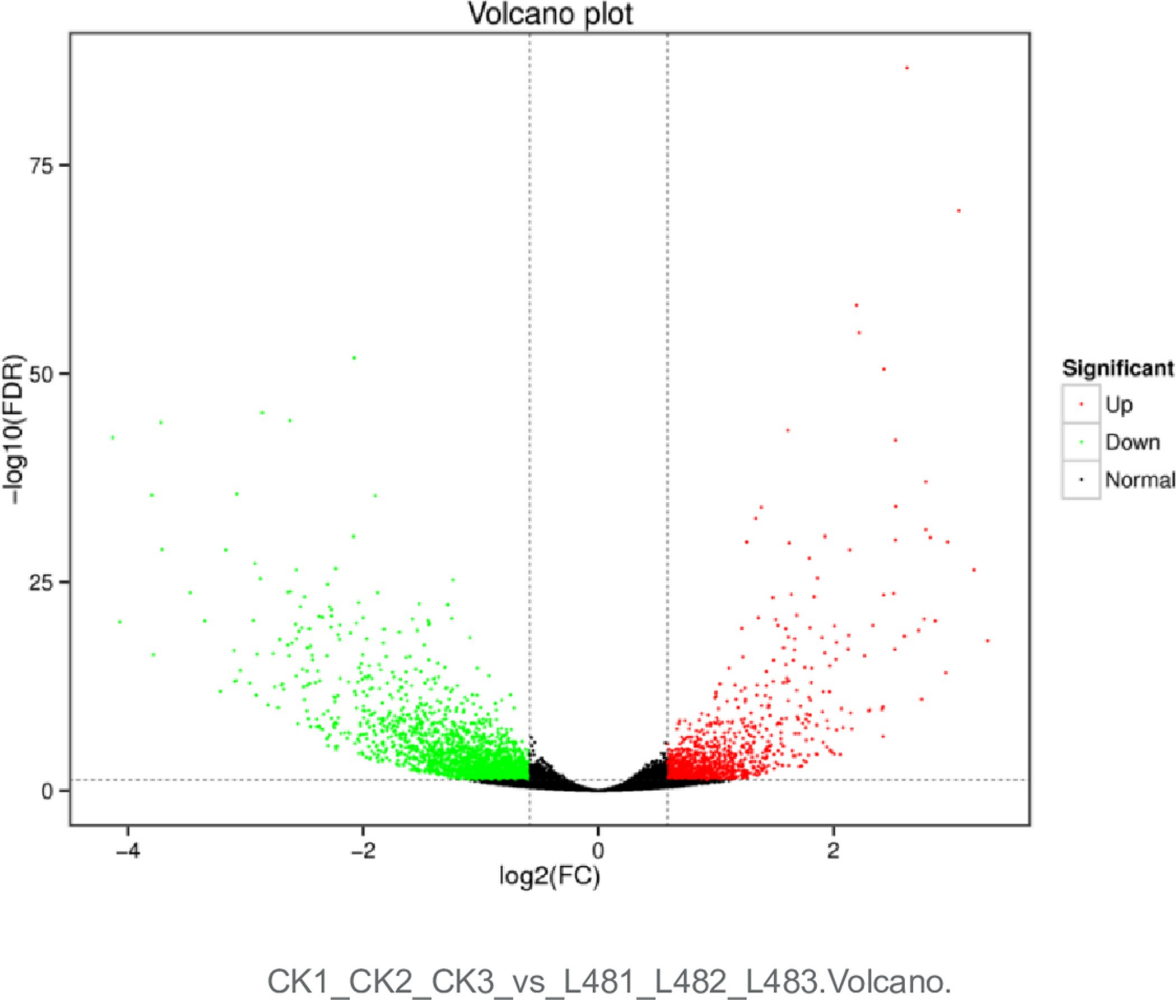
Volcano map of the differential genes. Each point in the differential expression volcano map represents a gene, and the horizontal coordinate represents the logarithmic value of the expression difference multiple of a certain gene in two samples. The ordinate represents the negative value of the statistical significance of the change in gene expression. The greater the absolute value of the horizontal coordinate, the greater the difference of the expression multiple between the two samples. The larger the ordinate value, the more significant the differential expression, and the more reliable the differentially expressed genes. The green dots represent down-regulated differentially expressed genes, the red dots represent up-regulated differentially expressed genes, and the black dots represent non-differentially expressed genes.

The transcriptome analysis of nine samples was completed, and a total of 65.33 Gb of clean data was obtained.We have uploaded the transcriptome data to the Gene Expression Omnibus (GEO) with the GSE number PRJCA026512. The clean data of each sample reached 6.86 GB, and the percentage of Q30 bases was 94.43% and above. The clean reads of each sample were sequenced with the designated reference genome (DMv6.1). Based on the comparison results, alternative splicing prediction analysis and gene structure optimization analysis were carried out to discover new genes. A total of 2624 new genes were discovered, of which 1565 were functionally annotated. Based on the comparison results, the gene expression was analyzed. The differentially expressed genes were identified according to the expression of genes in the different samples, and their functions were annotated and analyzed. There were 3236 differentially expressed genes in the potato variety "Atlantic" under 0 h and 48 h low light treatment, of which 1167 were up-regulated and 2069 were down-regulated(Fig 1).

#### 3.1.2 GO enrichment analysis of differentially expressed genes

**Fig 2:**
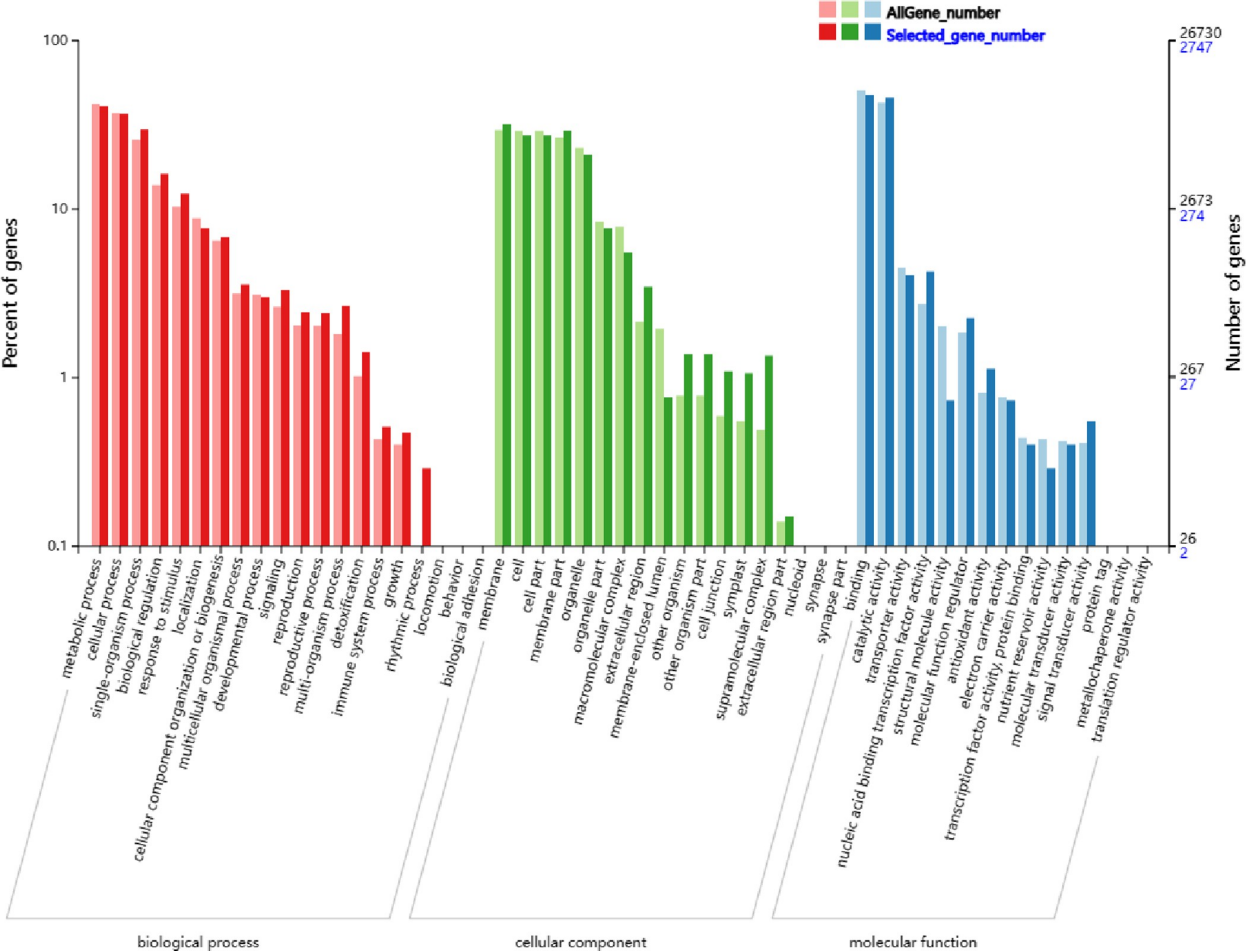
GO annotation analysis of differentially expressed genes. GO enrichment analysis of DEG of potato variety "Atlantic Ocean" under 0 h and 48 h low light treatment was carried out.

GO enrichment is divided into three primary units, namely biological processes, cellular components, and molecular functions. GO enrichment analysis of the differentially expressed genes of the potato variety "Atlantic" under 0 h and 48 h low light treatment was conducted. The results showed that the biological process has 20 secondary units, the cellular component has 18 secondary units, and the molecular function has 15 secondary units. In biological processes, differentially expressed genes were mainly concentrated in secondary units such as metabolic processes, cellular processes, single biological processes, biological regulation, response to stimuli, localization, cell component tissue or synthesis, and multiple biological processes. In cell composition, the differentially expressed genes were mainly concentrated in secondary units such as the membrane, cell, cell part, membrane part, and organelle. In molecular functions, differentially expressed genes were mainly concentrated in secondary units such as binding, catalytic activity, and transport activity (Fig 2).

#### 3.1.3 Enrichment analysis of KEGG pathway of differentially expressed genes

**Fig 3:**
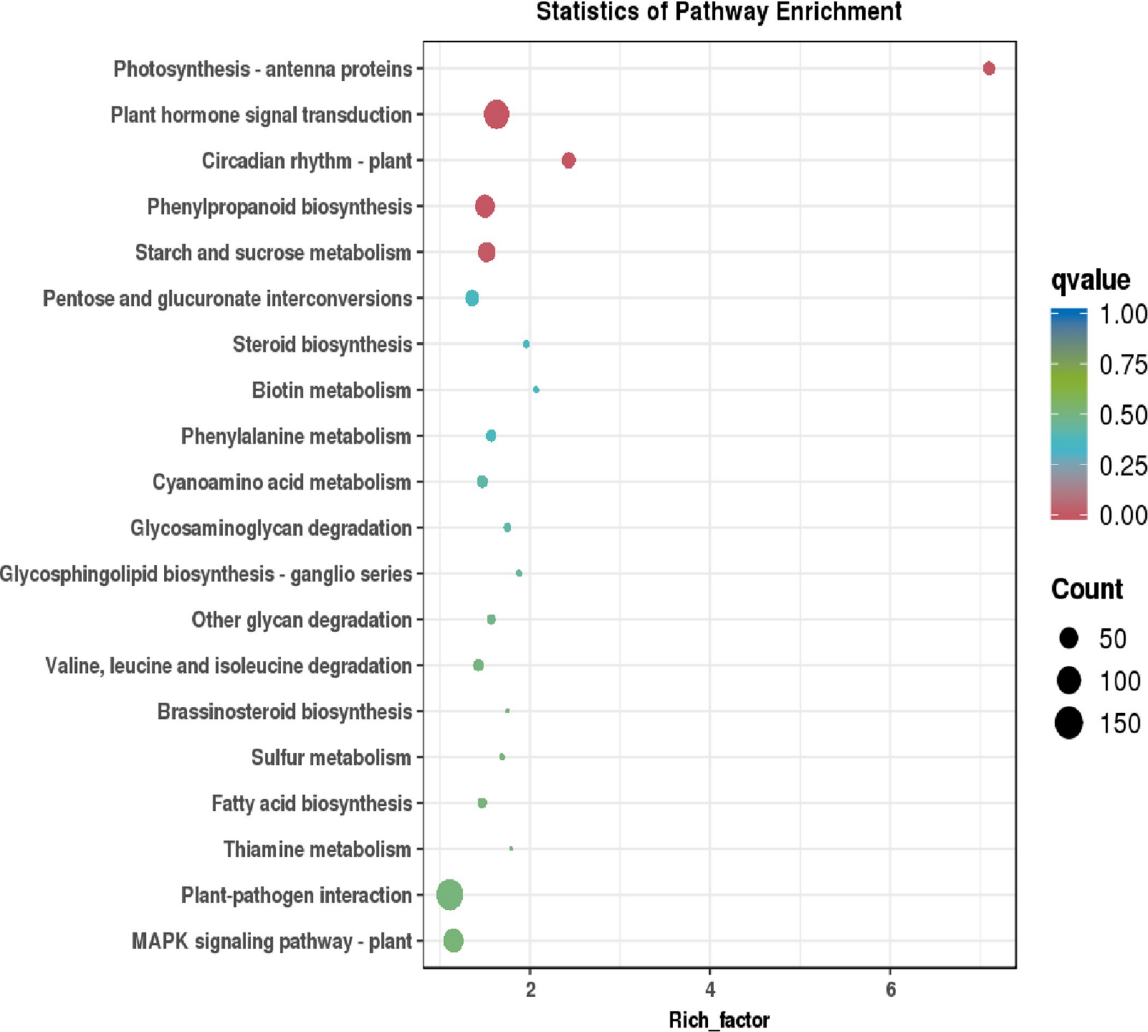
KEGG functional enrichment analysis of differentially expressed genes. The abscissa represents the enriched factor corresponding to each path, and the ordinate represents the path name. The color of the point reflects the p-value size. The redder the point, the more significant the enrichment. The size of the dot represents the number of enriched differential metabolites.

The differentially expressed genes of the potato variety "Atlantic" under 0 h and 48 h low light treatment were analyzed using KEGG pathway enrichment analysis, and 832 unigenes were annotated. The metabolic pathways enriched by these differential genes were the light-harvesting pigment-binding protein, plant hormone signal transduction, circadian rhythm, plant phenylpropanoid synthesis, and starch and sucrose metabolism. Among them, the number of differential genes in plant hormone signal transduction was the largest, the enriched factor in the light-harvesting pigment-binding protein pathway was the largest, and the enrichment degree was the highest (Fig 3). The light-harvesting pigment-binding protein genes play important roles in the differential genes related to the low light tolerance of the potato variety "Atlantic".

#### 3.1.4 Mining of genes related to low light tolerance in potato variety "Atlantic"

Among the differentially expressed genes, 27 differentially expressed genes were screened, including 26 up-regulated genes and 1 down-regulated gene(S1 Table in S1 Data). Twelve *Stlhcb* genes with high expression were selected from the twenty-seven genes for bioinformatics analysis and RT-qPCR verification (S2 Table in S1 Data).

### 3.2 Results of bioinformatics

#### 3.2.1 Analysis of physicochemical properties of potato light-harvesting protein

The primary structures of 12 light-harvesting pigment-binding proteins were predicted (S3 Table in S1 Data). The 12 light-harvesting pigment-binding proteins had between 3358 and 4852 atomic number, their relative molecular weight was between 24060.16 and 34624.54Da, and their PI was between 4.99 and 8.65. The total number of negatively charged residues (Asp + Glu) was between 22 and 33, and the total number of positively charged residues (Arg + Lys) was between 18 and 27. The instability coefficients of the 12 light-harvesting pigment-binding proteins were all less than 40, which indicates that they are predicted to be stable proteins. CP24, CP1B, LHCB1, CP40, and LHCB13 are hydrophilic proteins. LHCA4, CP8, CP50, LHCA3, LHCB12, CP3C, and CP5 are hydrophobic proteins.

#### 3.2.2 Prediction of secondary and tertiary structure of light-harvesting pigment-binding proteins

**Fig 4:**
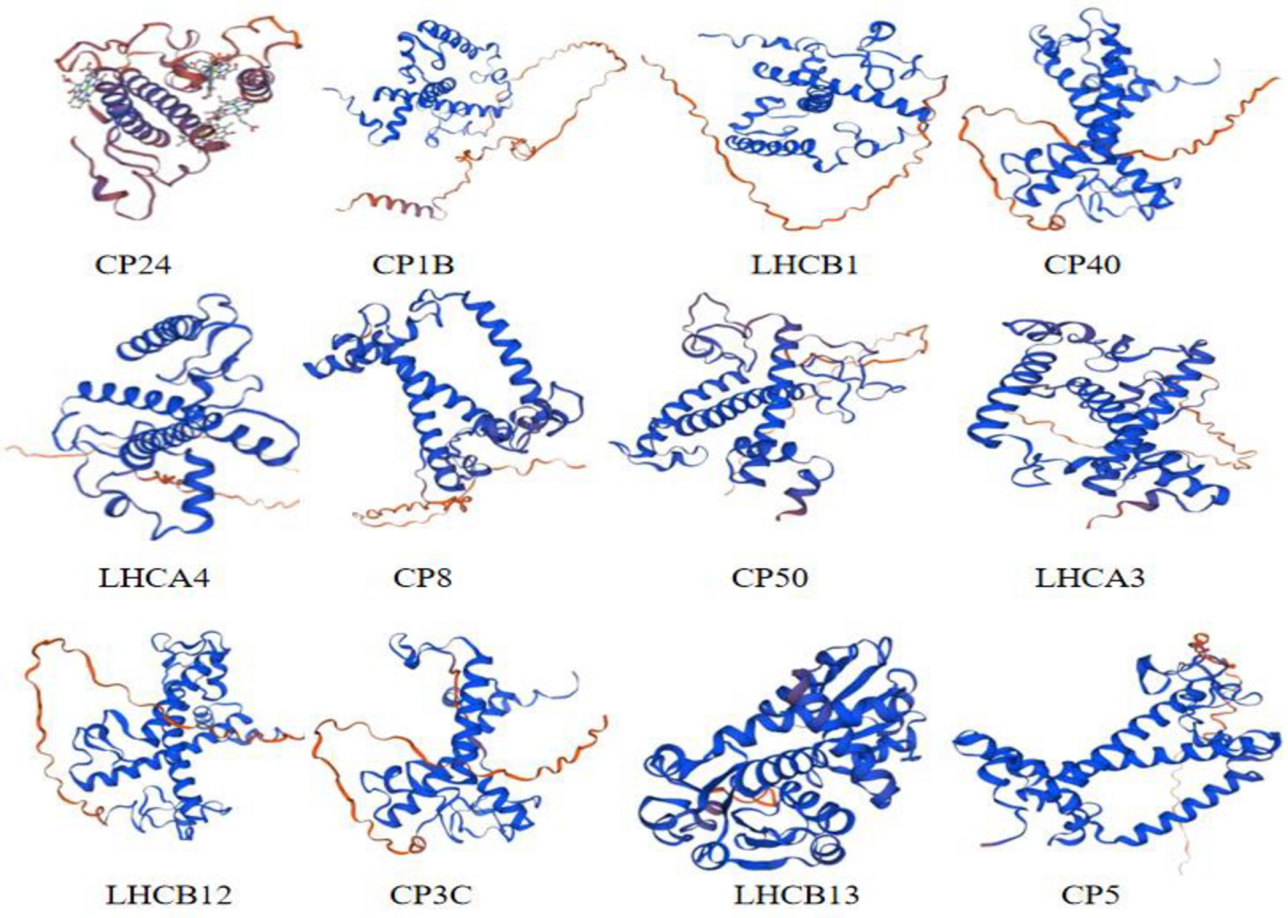
Prediction of the tertiary structure of 12 potato light-harvesting proteins. Spirals represent helices, broad strips with arrowheads represent β-pleated sheets, and thin loops represent random coils.

A prediction of the secondary structure of the 12 light-harvesting pigment-binding proteins was conducted using SOPMA. Among the 12 light-harvesting pigment-binding proteins, 106–156 amino acids participated in random coil, 78–110 amino acids participated in alpha helix, 9–47 amino acids participated in extended strand, and 4–18 amino acids participated in the beta turn. Thus, it can be seen that the largest element of the secondary structure of the 12 proteins is random coil (S4 Table in S1 Data). The tertiary structures of the 12 light-harvesting pigment-binding proteins were predicted using SWISS-MODEL (Fig 4). The results showed that the spatial structures of the 12 proteins were mainly random coil and α-helix, which is consistent with the prediction and analysis of the secondary structures.

#### 3.2.3 Prediction of subcellular localization of light-harvesting pigment-binding protein

We predicted the subcellular localization of 12 light-harvesting pigment-binding proteins in potato. The prediction was that these proteins are mainly located in the chloroplast and on the extracellular and plasma membranes. Light-harvesting pigment-binding proteins are mainly distributed on the thylakoid membrane of chloroplasts and participate in the light response of photosynthesis (S5 Table in S1 Data).

#### 3.2.4 Phylogenetic tree of light-harvesting pigment-binding protein

**Fig 5:**
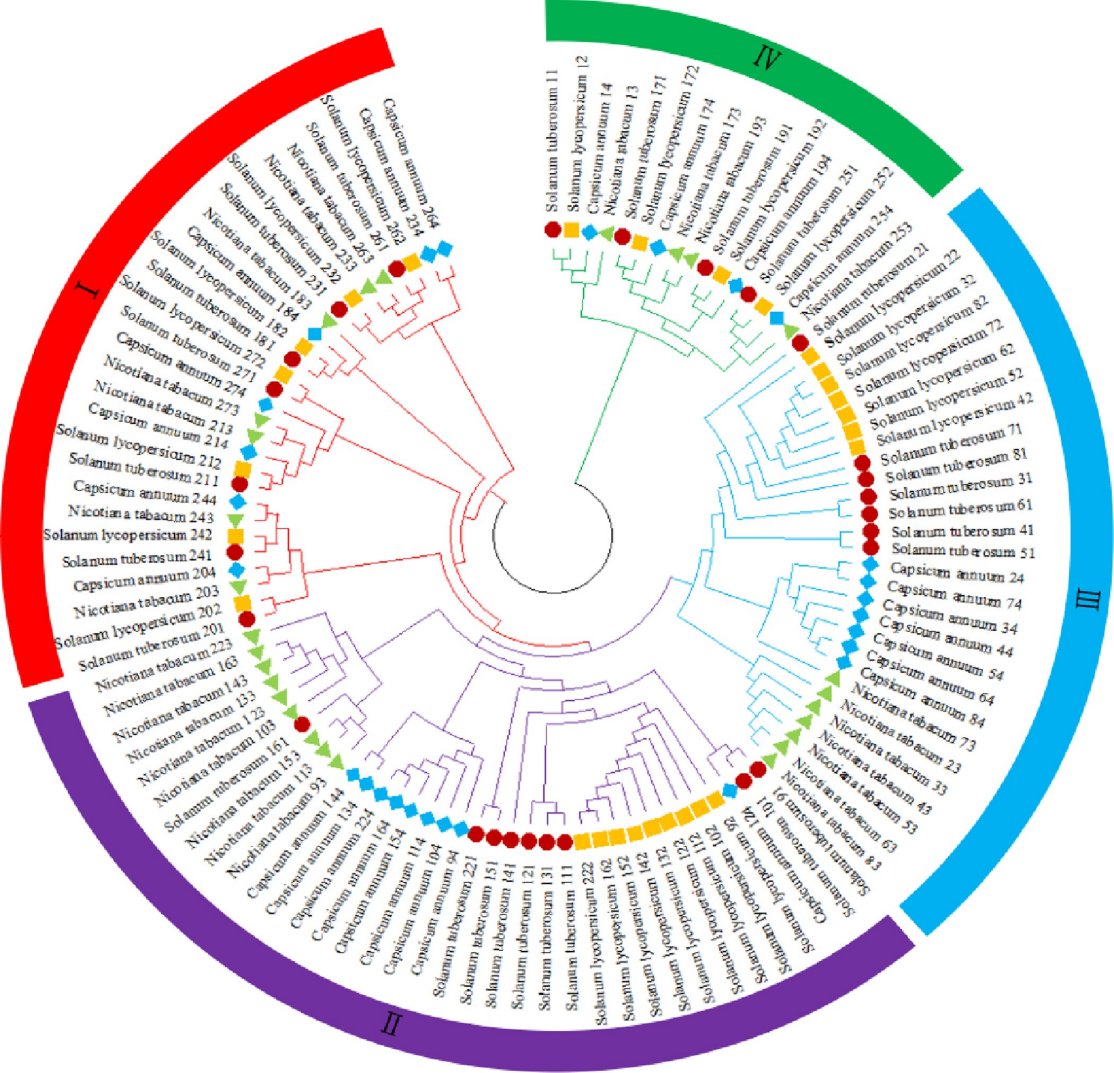
Potato light-harvesting pigment-binding protein phylogenetic tree. The red circles represent the potato genes. The yellow squares represent the tomato genes. The green triangles represent the tobacco genes. The blue diamonds represent the pepper genes.

The phylogenetic trees of the light-harvesting pigment-binding proteins in potato, tomato, tobacco, and pepper were constructed using the maximum likelihood method. The phylogenetic trees were divided into four subgroups (Fig 5). The phylogenetic results showed that the members of the light-harvesting pigment-binding protein family in potato are closely related to those of tomato.

### 3.3 Results of real-time fluorescence quantitative PCR

**Fig 6:**
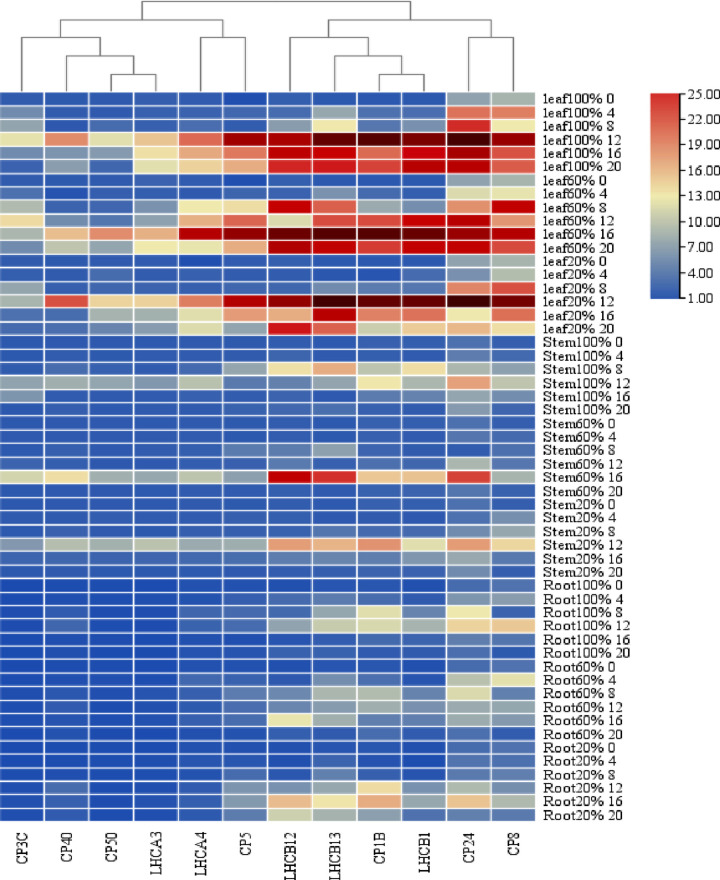
Heat map of time- and space-specific expression of *Stlhcb* genes. The expression levels of 12 *Stlhcb* genes in roots, stems, and leaves at 0 h, 4 h, 8 h, 12 h, 16 h, and 20 h under 100%, 60%, and 20% light conditions. The darker the color, the higher the expression.

The RT-qPCR results showed that the relative expression of the *Stlhcb* genes in the leaves, stems, and roots increased significantly under 60% (34 μmol·m^-2^·s^-1^) and 20% (11 μmol·m^-2^·s^-1^) weak light conditions (Fig 6). Under the 100%, 60%, and 20% light treatments, the relative expression of the *Stlhcb* genes increased first and then decreased with the extension of the light exposure. The relative expression of the 12 *Stlhcb* genes in the leaves was higher than that in the roots and stems. Under 60% light, the relative expression of the 12 *Stlhcb* genes in the leaves, stems, and roots increased by 1.0–6.8 times at 4 h, 1.1–12.3 times at 8 h, 1.9–26.5 times at 12 h, 2.1–36.3 times at 16 h, and 1.1–24.1 times at 20 h. Under 20% light, the relative expression of the 12 *Stlhcb* genes in the leaves, stems, and roots increased 1.0–3.7 times at 4 h, 1.4–5.5 times at 8 h, 1.7–31.2 times at 12 h, 2.8–22.3 times at 16 h, and 1.6–18.8 times at 20 h. Under the condition of 60% light for 16 h, the expression of the *Stcp24* gene was 33.4 times higher than that of the control. Under the condition of 20% light for 16 h, the expression of the *Stcp24* gene was 31.2 times higher than that of the control. The relative expression of the *Stlhcb* genes in the leaves was higher than that in the stems and leaves. Low light treatment could increase the relative expression of the *Stlhcb* genes, and the relative expression of the *Stcp24* gene was the highest among the 12 *Stlhcb* genes.

#### 3.4 Subcellular localization of light-harvesting pigment-binding protein gene *Stcp24*

**Fig 7:**
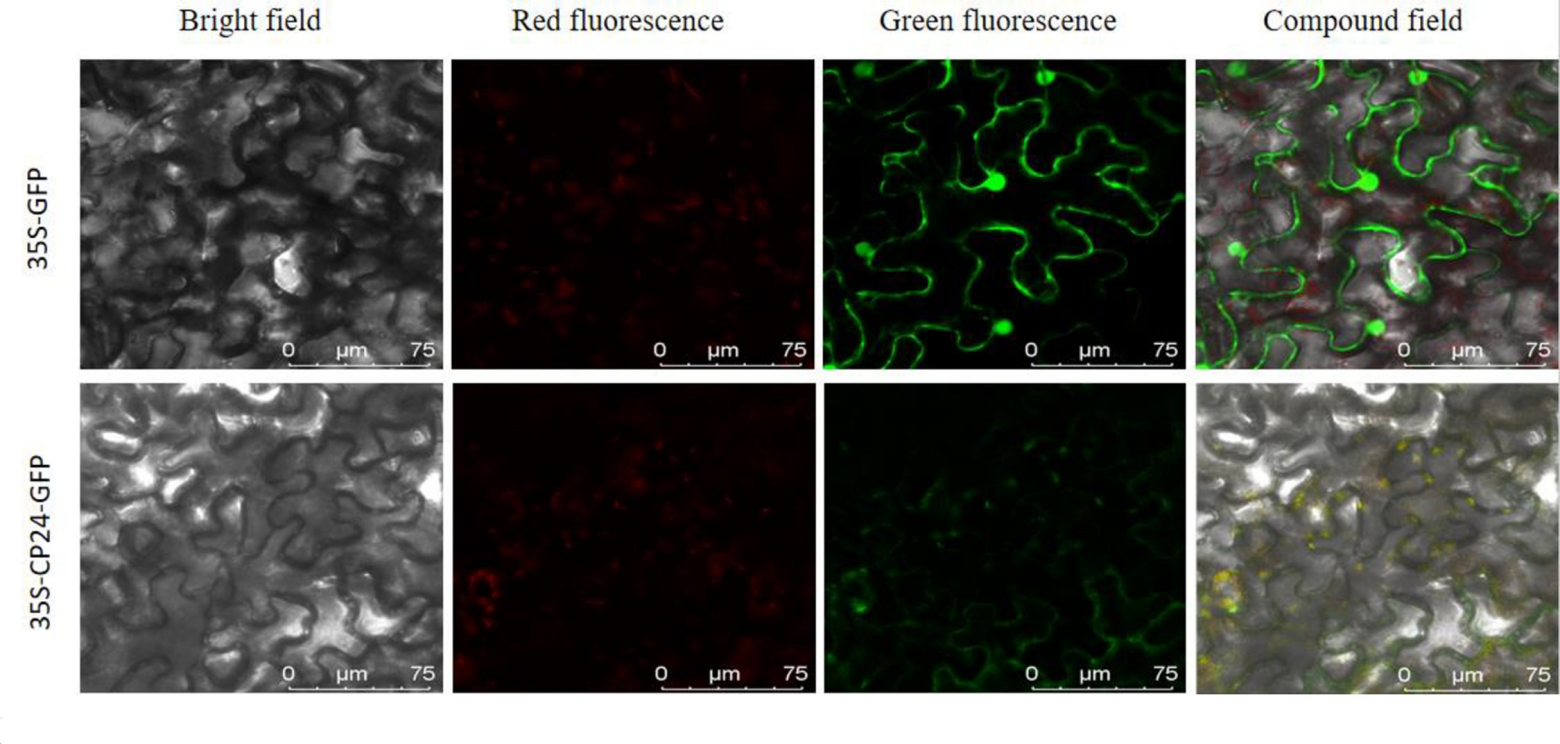
Transient expression of light-trapping pigment-binding protein gene *Stcp24*. The yellow in the compound field indicates chloroplasts.

After confirmation of its sequence, the pCAMBIA1300-CP24-GFP vector was transferred to Ben’s tobacco leaf by Agrobacterium. The empty vector pCAMBIA1300-GFP was used as a control. A confocal laser scanning microscope (TCS SP8 Leica Germany) was used to detect the GFP fluorescence signal at 488 nm wavelength. The results showed that the green fluorescent protein signal of the control group was distributed in the cell membrane, cytoplasm, and nucleus of the tobacco epidermis, but the green fluorescence signal of the fusion protein of CP24 and GFP was mainly concentrated in the chloroplast; therefore, the potato light-harvesting pigment-binding protein gene *Stcp24* was located in the chloroplast (Fig 7).

### 3.5 Cloning and vector construction of light-harvesting pigment-binding protein gene *Stcp24*

**Fig 8:**
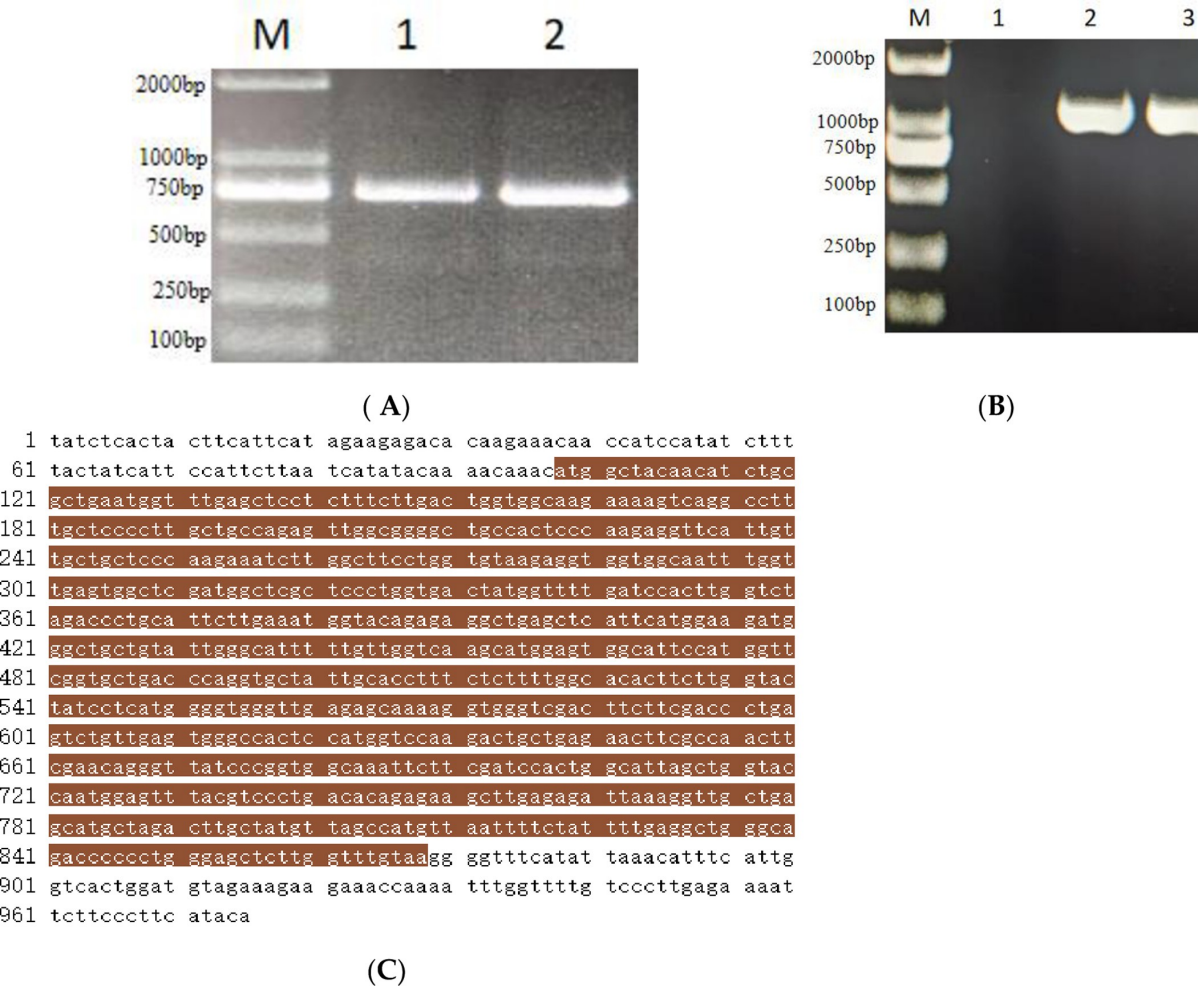
Cloning and linking of genes. (A) Electrophoretic amplification of *Stcp24* gene. M is the size marker; 1 and 2 are the target bands. (B) Electrophoresis of recombinant plasmid amplification. M is the marker; 1 is pCAMBIA1300; 2 and 3 are pCAMBIA1300-CP24. (C) *Stcp24* gene sequence. The brown part is the CDS sequence of the *Stcp24* gene.

The potato *Stcp24* gene fragment amplified by PCR was ligated into the TA/Blunt-zero clone vector and transferred into *E*. *coli* competent cells. The single colony resistant to kanamycin was verified using PCR (Fig 8A). After confirmation of its sequence, the plasmid was digested by BamHI and XbaI, and the target fragment was recovered and ligated to the plant overexpression vector pCAMBIA1300. This plasmid was transferred into *E*. *coli* and the plasmid was extracted (Fig 8B). The sequencing results showed that the cloned sequence was consistent with the sequence of the *Stcp24* gene published by NCBI (Fig 8C).

### 3.6 Identification of transgenic plants

**Fig 9:**
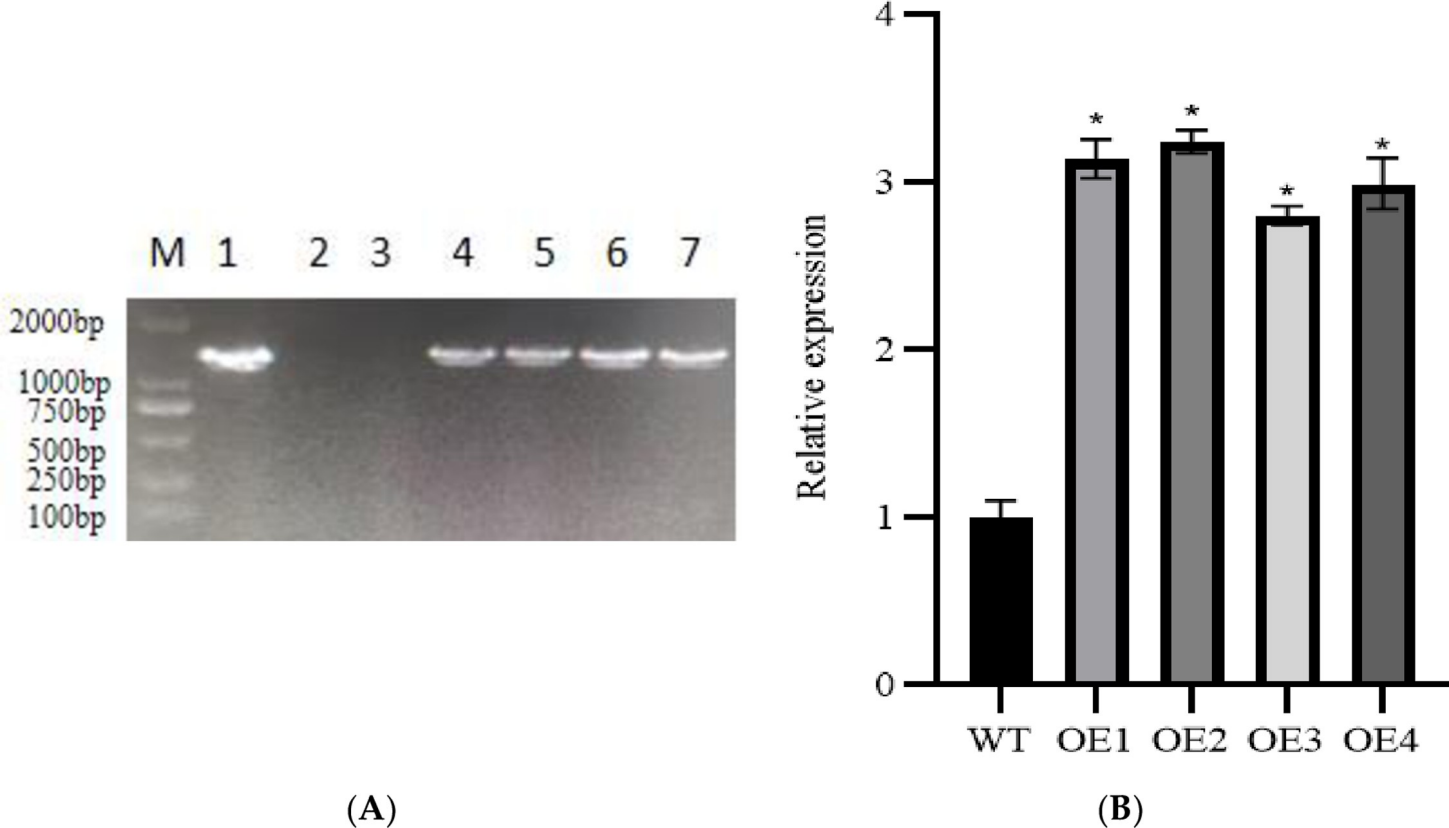
Verification of transgenic plants. (A) DNA amplification electrophoresis of transgenic plant leaves. M is size marker 2000; 1 is positive control; 2 is non-transgenic plant as a negative control; 3 is ddH2O as another negative control; 4 to 7 are transgenic plant lines. (B) RT-qPCR results. WT is a non-transgenic plant of the potato variety "Dongnong 310"; OE1 to OE4 are the transgenic plants.

The coding sequence of the potato *Stcp24* gene was ligated into the plant overexpression vector pCAMBIA1300. This vector was used to create transgenic potato overexpressing *Stcp24*. With the non-transgenic plant "Dongnong 310" as the control, the DNA of the regenerated seedlings was detected using PCR, and the bands were detected using agarose gel electrophoresis (Fig 9A). The results showed that the specific bands of 1000 bp were obtained in the plasmid vector and positive lines, but not in the negative control.

A total of four transgenic lines were obtained and were named OE1, OE2, OE3, and OE4. The expression of the *Stcp24* gene in the transgenic potato was detected at the transcriptional level using RT-qPCR (Fig 9B). The expression of the *Stcp24* gene in the OE1, OE2, OE3, and OE4 leaves of the transgenic plants was 3.13, 3.24, 2.81, and 2.99 times higher than that of the non-transgenic plants. These results showed that the *Stcp24* gene was overexpressed in OE1, OE2, OE3, and OE4.

### 3.7 Determination of physiological indexes of the transgenic plants

**Fig 10:**
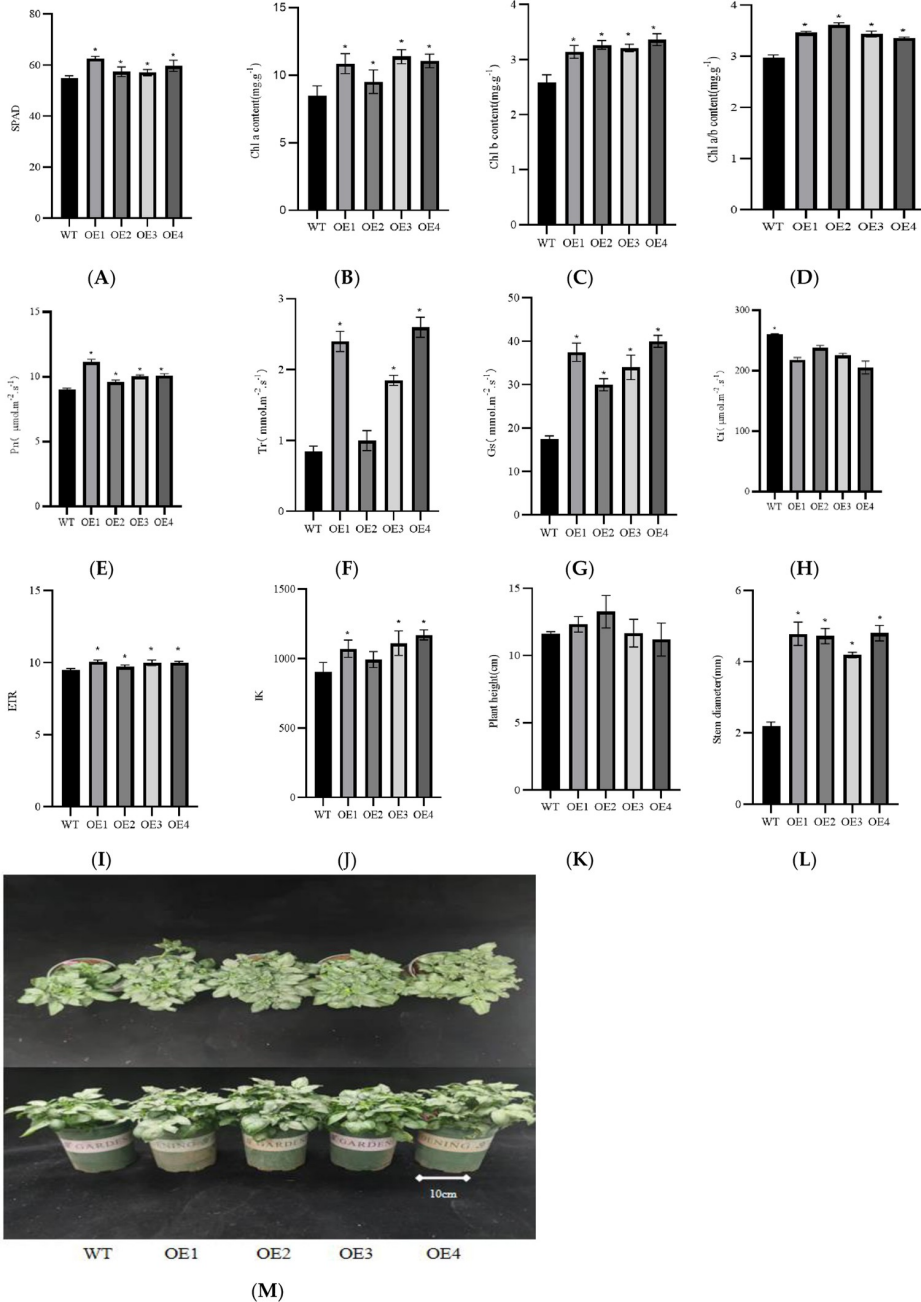
Physiological and morphological indexes of transgenic plants and non-transgenic plants. (A-D) SPAD of non-transgenic plants (WT) and transgenic plants (OE1, OE2, OE3, and OE4), chlorophyll a and chlorophyll b content and ratio. (E-H) Photosynthetic indexes net photosynthetic rate (*Pn*), transpiration rate (*Tr*), stomatal conductance (*Gs*), and intercellular CO_2_ concentration (*Ci*). (I-J) Fluorescence kinetic parameters electron transport efficiency (*ETR*) and half-saturation and light intensity (*IK*). (K-L) Plant height and stem thickness. (M) Transgenic plant and non-transgenic plant morphology.

The chlorophyll content analysis showed that the value of SPAD, the content, and the ratio of chlorophyll a and b in the transgenic plants were higher than those in the non-transgenic plants (Fig 10A–10D). The SPAD increased by 4.21%–14.44%; the chlorophyll a content increased by 12.53%–34.28%; the chlorophyll b content increased by 6.92%–14.11%; and the chlorophyll a/b increased by 12.79%–21.54%.

The analysis of the photosynthetic physiological parameters showed that compared with the non-transgenic plants, the net photosynthetic rate (*Pn*), transpiration rate (*Tr*), and stomatal conductance (*Gs*) of the transgenic plants increased by 6.41%–21.54%, 7.64%–27.33%, and 9.25%–30.8%, respectively (Fig 10E–10H). However, the intercellular CO_2_ concentration (*Ci*) decreased. *Pn*, *Tr*, and *Gs* were proportional to each other and inversely proportional to *Ci*. The fluorescence kinetic parameters showed that compared with the non-transgenic plants, the electron transfer efficiency (*ETR*) and half-saturated light intensity (*IK*) of the transgenic plants increased by 2.89%–16.66% and 6.22%–14.54%, respectively (Fig 10I–10J).

**Fig 11:**
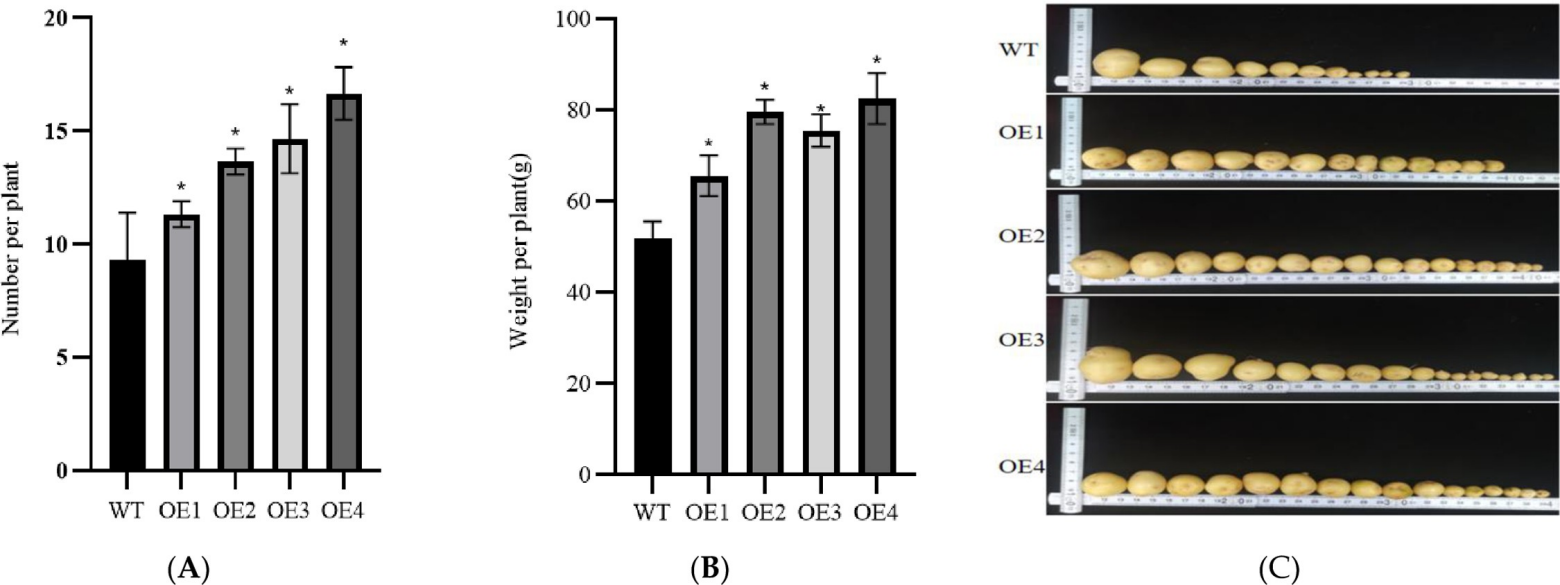
Potted plant potato yield. (A-B) Number and weight of potatoes per plant. (C) Yield per plant of transgenic plants and non-transgenic plants. WT is a non-transgenic plant of the potato variety "Dongnong 310"; OE1 to OE4 are transgenic plants.

In summary, these results showed that overexpression of the *Stcp24* gene in the transgenic potato increased the content and ratio of chlorophyll a and b in the leaves and increased *ETR* and *IK*; then, it increased *Pn*, *Tr*, and *Gs* and promoted plant growth. The plant height and stem diameter of the transgenic plants were significantly larger than those of the non-transgenic plants, reflecting the fact that the growth rate of the transgenic plants was higher than that of the non-transgenic plants (Fig 10K–10M). The potato yield per plant of the transgenic plants was higher than that of the non-transgenic plants. The potato yield of the OE2 and OE4 plants was higher than that of the non-transgenic plants (Fig 11A–11C).

## 4 Discussion

Light capture is an important process of plant photosynthesis. Light-harvesting pigment–protein complexes capture light energy and quickly transfer it to the reaction center [16]. Light-harvesting pigment-binding proteins have a variety of functions, including the capture and transfer of light energy or photochemical quenching under different levels of sunlight intensity in nature. Light-harvesting pigment–protein complexes are part of two different light energy conversion systems: photosystem **I** light-harvesting pigment-binding protein complex (LHC**I**) and photosystem **II** light-harvesting pigment-binding protein complex (LHC**II**). LHC**II** is composed of highly conserved Lhcb subtypes, and all green organisms contain a large number of *Lhcb* genes [17]. The six genes encoding LHC**II** were named LHC**II**b (*Lhcb1*, *Lhcb2*, and *Lhcb3*); LHC**II**a (*Lhcb4*, also known as *CP29*); LHC**II**c (*Lhcb5*, also known as *CP26*); and LHC**II**d (*Lhcb6*, also known as *CP24*) [18].

A lack of light-harvesting pigment-binding protein leads to a decrease in chlorophyll content in *Arabidopsis thaliana* and a light green leaf color [19]. The members of the light-harvesting pigment-binding protein family play an important role in plant adaptation to environmental stress. Down-regulation of the light-harvesting pigment-binding protein family members can reduce the tolerance of plants to environmental stress and reduce seed yield [20]. Xu et al. have shown that the down-regulation of LHCB family members reduces the tolerance of *A*. *thaliana* to drought stress [21]. Minagawa et al. studied the structure and function of light-harvesting pigment-binding protein in photosystem **II** and pointed out that the *LHCb* gene encodes the LHC**II** protein, which is the main light-harvesting chlorophyll a and b binding protein of photosystem **II**. Among the three LHC**II** proteins, Lhcb1 and Lhcb2 are the most abundant [22]. When the expression of Lhcb1 and Lhcb2 is inhibited, the subunit of the LHC**II** complex is not synthesized [23].

Boekema et al. found that the photosynthetic rate and growth rate of plants lacking CP24 decreased under photoinhibition in *A*. *thaliana* [24]. CP24 plays an important role in the structure and function of the PS**II** light-harvesting pigment-binding protein [25]. CP24 is a trace light-harvesting pigment-protein complex of photosystem **II** and is a unique antenna complex of terrestrial plants. Recently, some studies have shown that a certain number of chlorophyll and other pigment molecules bind to CP24 protein to maintain its stability and activity.

Mishanin et al. reported the effect of light on chlorophyll accumulation, and subsequent studies showed that chlorophyll accumulation increased under low light conditions [26]. Stolárik et al. showed that the absorption spectra of different plant pigments were different and that a change in light intensity changed the pigment composition in LHC [27]. Croce et al. found that light-harvesting pigment-binding proteins collected photons under low light conditions [28].

The transcriptional expression of the *Lhcb2* gene in pea was controlled by light and was obviously dependent on the length of light exposure. The *Lhcb2* gene was not expressed under 0–1.5 h of light but was expressed under more than 2 h of light [29]. Under shading conditions, a shade-avoidance reaction occurred in soybean, and the photosynthetic capacity of the leaves decreased [30]. Ferroni et al. proved that Lhcb6 participates in the energy balance between PS**I** and PS**II** through a unique reversible phosphorylation process and also participates in the photosynthesis of stone pine plants [31].

Photosynthesis is an important metabolic process used by plants to convert sunlight energy into chemical energy and provide energy for themselves [32]. In the photoreaction stage, the light collected by photosynthetic pigment molecules such as chlorophyll drives the process of photosynthetic electron transport through PS**I** and PS**II** [33]. Kale et al. found that the expression levels of several genes in the PS**I** and PS**II** of *Alsophila spinulosa* leaves were significantly up-regulated under 10% shaded light [34]. Chen et al. found that 40% shading treatment significantly up-regulated the expression of the PsaB, PsaD∼H, PsaK, PsaL, PsaN, PsaO, PsbA, PsbO, PsbP, PsbS, and PsbW genes in PS**I** and PS**II** [35]. In this study, it was also found that the expression of the 12 light-harvesting pigment-binding protein genes in PS**II** increased significantly under 20% and 60% light.

Johnson et al. found that tobacco leaf shading can upregulate some genes in the light system and that plants achieve effective photosynthesis through light capture in order to adjust the energy flow balance and improve the expression level of photosystem-related genes under low light conditions [36]. In the study by Xue et al., it was found that short-term weak light could increase the expression of light-harvesting pigment-binding protein genes [37]. Long-term low light causes light fluctuation and destroys the energy flow balance of PS**I** and PS**II** in plants. The expression of genes related to photosynthetic electron transport is down-regulated under chronic low light.

The leaf photosynthetic pigment–protein complex is an important basis for plant photosynthesis; it can not only absorb, transfer, and transform light energy, but also adapt to the changes in the external light environment by adjusting its content and structure [38]. Weak light is generally understood to be an ambient light intensity that is significantly lower than the light saturation point, but not lower than the lowest light intensity that limits survival; it can be called weak light stress [39]. The cost of energy (light) accounts for a large proportion of the total expenditure in the production of virus-free potato tissue culture seedlings [40]. Low light-resistant varieties can reduce energy consumption in potato tissue culture, and it is thus desirable to cultivate varieties that are resistant to low light.

In this experiment, 56 μmol·m^-2^·s^-1^ light was selected as the control, and 34 μmol·m^-2^·s^-1^ and 11 μmol·m^-2^·s^-1^ light treatments were set up as low light treatments. Some studies have found that chloroplasts are mainly distributed in mesophyll cells and stem epidermis cells, where photosynthesis can be carried out [41]. In this study, the expression of 12 light-harvesting pigment-binding protein genes under low light treatment showed obvious spatio-temporal specificity. Their expression levels peaked at 12–16 h of light and then began to decline, but the 20 h value was still higher than the initial value. Their expression level in the leaves was also significantly higher than that in the stems and roots. The light-harvesting pigment-binding protein genes are mainly expressed in chloroplasts, and chloroplasts are mainly present in leaves.

In this study, we analyzed the amino acid composition, hydrophilicity, and secondary and tertiary structures of 12 light-harvesting pigment-binding proteins. The results showed that CP24, CP1B, LHCB1, CP40, and LHCB13 were hydrophilic proteins. LHCA4, CP8, CP50, LHCA3, LHCB12, CP3C, and CP5 were hydrophobic proteins. The results of the subcellular localization experiment showed that the CP24 protein was mainly located in the chloroplast, which was consistent with the predicted function of the protein. However, in the subcellular localization image, we can see that a small amount of the green fluorescent protein gene is located on the cell membrane and that a small amount of chloroplast is not stained, which may be due to the failure of *Agrobacterium tumefaciens* to completely infect all the cells, or it may be due to the long post-injection treatment time. Previous studies have shown that in general, protein expression occurs after 24 h of transformation, but gradually disappears after 48 h [42].

The light-harvesting pigment-binding protein gene family is closely related to chlorophyll content and plant photosynthesis [11]. The chlorophyll a/b value in the transgenic plants overexpressing *Stcp24* was higher than that in the non-transgenic plants; so, the transgenic plants may be better able to absorb red light and adapt to light deficiency conditions. Among the photosynthetic indexes, the net photosynthetic rate, transpiration rate, and stomatal conductance were directly proportional to the intercellular CO_2_ concentration. Xiao et al. found that an increase in light-harvesting pigment-binding protein increased the accumulation of chlorophyll [43]. In this experiment, the overexpression of the *Stcp24* gene combined with more chlorophyll a and b increased the content of chlorophyll.

Zou et al. found that light-harvesting pigment-binding proteins have functions related to the aggregation and binding of pigments and the transferal of electrons [44]. Pigments gather and bind to light-harvesting pigment-binding proteins to maintain pigment activity and a measurable state. In this experiment, the overexpression of the *Stcp24* gene increased the electron transfer efficiency and semi-saturated light intensity. Exposure of lettuce to higher light intensities (non-stress threshold levels) resulted in higher biomass accumulation, faster photosynthetic phylogenesis, and better water use efficiency [45].The photosynthetic performance of cut roses was better under the supplementary light with high red light ratio, which was conducive to the improvement of growth condition and yield [46].These plant adaptations can be induced by morphological modifications [47]. For instance, exposure to low light intensities can lead to stem and leaf elongations and other morphological modifica-tions to maximize absorption of the available light to meet the demand for photosynthesis [48], while exposure to high light intensities can cause plant compact-ness and reduction in leaf area expansion to decrease energy absorption in response to elevated irradiance [49]. Exposure to high light intensities can result in photoinhibition [50]; CP24 can form a complex with zeaxanthin, which significantly reduces chlorophyll content in leaves. Under low light conditions, the content of chlorophyll b is increased to adapt to the conditions with fewer photons [31].

Mishanin et al. found that the light-harvesting pigment-binding proteins CP24 and CP29 also played an important role in regulating photosynthesis. The photosynthetic rate and growth rate of plants lacking CP24 decreased significantly [51]. In this experiment, the overexpression of the light-harvesting pigment protein gene *Stcp24* increased the net photosynthetic rate and plant height and stem diameter. The overexpression of the light-harvesting pigment protein gene *Stcp24* increased the expression of the CP24 protein and then increased the content of chlorophyll a and chlorophyll b. Therefore, the values of chlorophyll a and b and SPAD increased. The increase in the chlorophyll a and b content enables the plant to absorb more light energy for the light response stage of photosynthesis. Chlorophyll absorbs light energy and is directed to the reaction center to transfer energy to low-energy electrons and convert them into high-energy electrons [52]. Therefore, the *ETR* and *IK* of the plant increased. High-energy electrons are transferred to thylakoid membrane proteins and bind to ADP through the electron transport chain to form ATP. ATP participates in the synthesis of sugars with CO_2_ in the dark reaction [53]. Therefore, the *Pn*, *Tr*, and *Gs* of the plant increased, and the *Ci* decreased. The increase in the net photosynthetic rate of the plant causes the accumulation of more organic matter, which improves the growth of the plant. Therefore, increased plant height and stem diameter resulted in an increased potato yield per plant. In summary, the growth state of the transgenic plants was better than that of the non-transgenic plants.The increase of gene expression of light-harvesting pigment binding protein in potato pathway can increase chlorophyll content, enhance the ability of chloroplast to capture light energy, increase the number of transferred electrons, enhance the ability of carbon fixation in dark reaction, and increase the synthesis of organic matter.

## 5 Conclusions

Potato is an important food crop, and it is particularly important to enhance potato photosynthesis and thereby increase the potato yield. The light-harvesting pigment-binding protein complex is very important for plant photosynthesis. Through transcriptome differential gene analysis and bioinformatics analysis, we found that the 12 *Stlhcb* genes played an important role in potato tolerance to low light. We showed that the *Stlhcb* genes had tissue- and time-specific expression in potato. The expression of the *Stlhcb* genes in leaves was significantly higher than that in the stems and roots, and the expression first increased and then decreased over time. *Stcp24* with the highest expression was cloned for subcellular localization and stable genetic transformation experiments. The subcellular localization on the chloroplast was consistent with the predicted results, and transgenic plants overexpressing *Stcp24* had a higher yield of potatoes. In summary, the results presented in this study demonstrate that overexpression of the *Stcp24* gene in the potato increases the content of chlorophyll a and b, which improves the efficiency of electron transfer and photosynthesis to promote plant growth.

## Supporting information

S1 Data Supplementary material to the article. (XLSX)

S1 Raw image (PDF)
