# Supplementary material for: Expression pattern of Stlhcb gene family in potato and effects of overexpression of Stcp24 gene on potato photosynthesis
Source: PLoS One. 2024 Aug 23;19(8):e0305781. doi: 10.1371/journal.pone.0305781 (PMC11343382; doi:10.1371/journal.pone.0305781)
Supplement: S1 Raw image — (PDF) [file pone.0305781.s002.pdf]

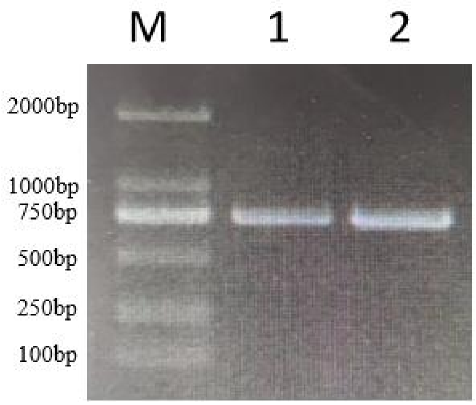

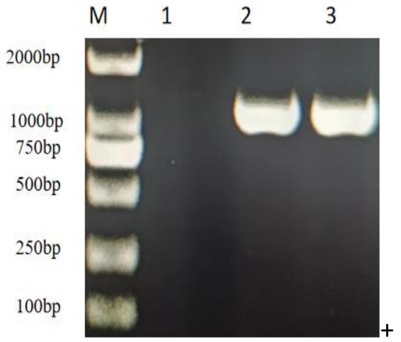

(A) PCR band of *CP24* gene. M is maker; 1 and 2 are the target bands. (B) Recombinant plasmid PCR bands. M is maker; 1 is empty plasmid; 2 and 3 were recombinant plasmids.

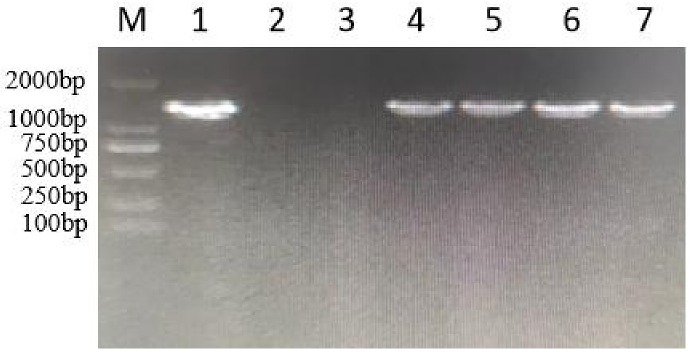

DNA amplification electrophoresis of transgenic plant leaves. M is maker2000, 1 is positive plasmid, 2 is non-transgenic plant strip, 3 is ddH_2O, 4 to 7 are transgenic plant strips.
